# Math anxiety and deficient executive control: does reappraisal modulate this link?

**DOI:** 10.1111/nyas.14772

**Published:** 2022-04-07

**Authors:** Lital Daches Cohen, Orly Rubinsten

**Affiliations:** ^1^ Edmond J. Safra Brain Research Center for the Study of Learning Disabilities, Department of Learning Disabilities University of Haifa Haifa Israel

**Keywords:** math anxiety, emotion regulation, reappraisal, suppression, executive control

## Abstract

The literature suggests an interplay between executive control functions and emotion regulation processes, with each playing a key role in math anxiety. We examined the relation between the use of two different emotion regulation strategies (reappraisal and suppression) and the ability to reduce emotional interference in high‐conflict situations (i.e., executive control of attention) in cases of math anxiety. A sample of 107 adults completed emotion regulation tendencies and math anxiety questionnaires and performed a flanker task following the priming of a math‐related or negative word. The findings revealed: (1) highly math‐anxious individuals had difficulty controlling emotional distractions induced by math information, even as simple as math‐related words, in high‐conflict conditions; and (2) the tendency to use reappraisal in everyday situations was associated with math‐anxious individuals’ ability to avoid heightened emotional reactions when encountering math‐related (i.e., threatening) information. These findings point to the efficacy of reappraisal‐focused intervention and suggest an innovative mechanism through which reappraisal reduces emotional reactions and improves performance among math‐anxious individuals, indicating a new way to approach interventions for math anxiety.

## Graphical abstract

In this study, we examined the relation between the use of two different emotion regulation strategies (reappraisal and suppression) and the ability to reduce emotional interference in high‐conflict situations (i.e., executive control of attention) in cases of math anxiety.

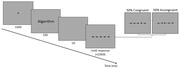

## Introduction

Math anxiety is a common phenomenon^1^ characterized by negative attitudes to math,^2^, ^3^ including stress, frustration, and fear when thinking about or engaging in number manipulation and mathematical problem‐solving.^3^, ^4^, ^5^ For math‐anxious individuals, mathematical information is perceived as a threatening stimulus^3^, ^6^, ^7^ and evokes attentional disengagement, even when briefly presented.^7^, ^8^ Due to this math‐based emotional interference,^9^, ^10^ both children^11^ and adults^12^, ^13^ show impaired executive control of attention, a high‐order cognitive operation that enables goal‐directed behavior by inhibiting irrelevant information.^14^, ^15^

Emotion regulation modulates the effects of emotion on executive control of attention.^16^, ^17^ It is a mental process that affects the type, duration, intensity, and expression of emotions.^18^, ^19^ Neuroimaging^20^ and behavioral data^21^, ^22^ suggest that executive control mechanisms and emotion regulation processes are interrelated and involved in the same brain regions. However, the literature on math anxiety has focused on either executive control^23^ or emotion regulation.^24^ We explored the relations between math anxiety, executive control of attention, and emotion regulation.

### Math anxiety

Math anxiety has far‐reaching consequences.^25^, ^26^ It is associated with poor math skills^27^ and reduced math‐related cognitive abilities,^3^, ^28^ regardless of working memory capacity or processing speed.^29^ In the long term, it is linked to the avoidance of math‐related careers,^3^, ^30^ increased health costs,^31^ reduced financial literacy,^32^ and low socioeconomic status.^33^ Despite the plethora of concerns, the mechanism underlying math anxiety is not well understood.^6^

According to attentional control theory,^34^, ^35^ similar to other anxiety syndromes, math anxiety impairs the functioning of goal‐directed cognitive systems (i.e., task‐orientated problem solving)^23^, ^36^, ^37^ by increasing attention to threat‐related information and intrusive thoughts.^27^ In one study, math‐anxious individuals demonstrated delayed cognitive processing, manifested by longer P3b latency during a numerical comparison task.^38^ A study using neural network modeling^6^ found that math anxiety was linked to increased activation in the amygdala and anterior cingulate cortex, leading to increased stress and greater perception of conflict. As a result of the stress perceptions in math anxiety, activation in the prefrontal cortex can be disrupted, affecting executive control processes and goal‐directed behavior.^39^ Recent findings, however, suggest that math anxiety has different, and even stronger, relations with higher cognitive structures, such as working memory, compared to general anxiety.^40^ Thus, it is possible that math anxiety has a specific association to executive control processes, which is different from the association of other anxiety syndromes to executive functions.

In the cognitive sciences, it is common to use tasks that activate opposing response options to measure and manipulate executive control of attention. In the flanker task,^41^ participants must respond to relevant information (e.g., a flanker target, such as an arrow in the center of the screen) and ignore irrelevant information (e.g., flanking arrows that surround the center arrow). In congruent trials, the relevant and irrelevant information require the same response (e.g., arrows point in the same direction), while in incongruent trials, opposite responses are needed (e.g., arrows point in the opposite direction). The conflict arising in incongruent trials requires the engagement of executive control, and this requirement is reflected in slower response time (RT).^17^

Highly math‐anxious individuals show a greater difference in RTs in incongruent versus congruent trials (i.e., congruency effect^42^) than slightly math‐anxious individuals,^37^ especially when interference cannot be anticipated,^23^ thus suggesting math anxiety is accompanied by difficulties in the ability of executive control to inhibit irrelevant and interfering information.^37^ Here, we focus on executive control (i.e., incongruent trials).

### Math anxiety and executive control of attention

Math anxiety has been linked to impaired executive control of attention.^11^, ^12^, ^13^, ^23^, ^36^, ^37^ Compared to slightly math‐anxious individuals, those with high math anxiety demonstrated significantly greater P300 amplitude^43^ and beta‐band power oscillation,^44^ as well as smaller gamma band activity^45^ when they anticipated arithmetic problems and greater gamma band activity when solving these problems.^13^

Executive control mechanisms may help attenuate emotional effects,^17^, ^46^, ^47^ possibly because emotions trigger attentional control, which enables enhanced conflict resolution.^42^, ^48^ With increased life stress, greater activity in the dorsolateral prefrontal cortex (the area supporting executive control^39^) has been associated with reduced anxiety symptoms.^49^

One possible strategy for studying the link between emotions and executive control is to use *emotional priming* tasks assessing implicit responses to an irrelevant emotional stimulus.^50^ People usually show slower RTs to task‐relevant goals following the priming of a stimulus with a negative valence,^51^ arguably because of the effect of emotional valence on information processing.^52^ Impairments in the ability of executive control to reduce these emotional distractions should be manifested in a greater difference between RTs following emotional and neutral priming in high‐conflict situations (i.e., incongruent trials in the flanker task). The size and characteristics of this difference in performance reflect the efficiency and limits of selectivity, and provide valuable information about the mechanisms involved in controlling the conflict.^17^

To date, no one has investigated the link between math anxiety‐related emotional effects (e.g., exposure to math‐related information as a prime before the task) and the executive control of attention. As math anxiety hampers executive control mechanisms,^6^, ^23^, ^36^, ^37^, ^38^ we innovatively examined executive control of attention in math‐anxious individuals during emotional interference (i.e., in incongruent trials) of stimuli with either negative valence or math‐based (presented as primes).

The literature suggests that impairments in executive control mechanisms may be related to difficulties in emotion regulation.^21^, ^22^, ^46^, ^47^, ^49^ Thus, we also manipulated and measured emotion regulation strategies.

### Linking emotion regulation and executive control of attention in math anxiety

The ability to adaptively regulate emotional experiences is necessary when confronted with threatening information.^53^ Two widely studied emotion regulation strategies are cognitive reappraisal and expressive suppression.^18^, ^54^ *Cognitive reappraisal* constitutes an antecedent‐focused strategy that aims to modify thoughts and beliefs about a stimulus or situation in a way that alters the emotional response. *Expressive suppression* is a response‐focused strategy in which the individual attempts to conceal his/her feelings, behaviors, and physiological activity.^18^ While the use of reappraisal leads to a decrease in the subjective experience of negative emotions^55^, ^56^, ^57^ and more adaptive behavioral,^55^, ^58^, ^59^ physiological,^58^, ^60^, ^61^ and neural responses^56^, ^59^ to emotionally evocative events, suppression is associated with decreased positive affect.^18^

The literature points to the central role of reappraisal in reducing math anxiety reactions^62^, ^63^, ^64^ and improving math performance.^24^, ^62^, ^64^, ^65^, ^66^, ^67^, ^68^, ^69^ These findings can be explained by the relations between reappraisal and reduced activation in emotion‐related brain regions (i.e., amygdala^70^) and increased activation in the fronto‐cingular network,^20^, ^49^ which is involved in domain‐general executive control.^71^

Reappraisal ability^21^, ^72^ and the frequency with which reappraisal is used in everyday situations^22^ have been linked to executive control of attention (indicated by the ability to reduce emotional interferences in incongruent situations). Similarly, executive control training has led to greater success in implementing reappraisal.^46^, ^47^

### The study

Research in the field of math anxiety usually examines separately emotional influences, such as emotion regulation,^64^ and cognitive factors, such as executive control of attention.^23^ Given the growing recognition of the interplay between cognitive and emotional processes,^73^ we explored the relations between the use of different emotion regulation strategies and the ability to reduce emotional interference in incongruent situations (i.e., executive control of attention) in cases of math anxiety.

Specifically, we examined: (1) the effects of emotional interference on the ability to reduce emotional interference as part of executive control of attention in math anxiety, while differentiating between stimuli with negative valence and math‐based emotional interference; and (2) the ability of emotion regulation to modulate the link between math anxiety and the (possible) deficient ability of executive control to reduce emotional distractions induced by math‐related stimuli.

In line with attentional control theory^34^ and evidence on the difficulties involved in exerting executive control in math anxiety,^23^, ^36^, ^37^ specifically the effects of math‐related stimuli on goal‐directed behavior,^9^, ^10^, ^38^ we hypothesized that mathematical information, perceived as threatening by math‐anxious individuals,^3^, ^6^, ^7^ would have more pronounced effects on performance in the incongruent trials in the flanker task than stimuli with negative valence. Unlike congruent trials, incongruent trials require the recruitment of the executive control of attention. Consistent with previous findings demonstrating relations between executive control of attention and reappraisal,^20^, ^21^, ^22^, ^46^, ^47^, ^49^ we hypothesized that frequent use of reappraisal would be linked to decreased emotional distractions (i.e., higher levels of executive control).

## Method

### Participants

We calculated sample size according to the previously reported correlations between math anxiety and executive control of attention. Based on *r* = −0.35,^37^ power = 0.8, and a significance level of 0.05, we concluded 46 participants would be sufficient.

Given the high dropout rates in Internet‐based research,^74^ the initial sample included 134 adults (95 females; *M* = 28.39 years, *SD* = 4.21). Six males and 10 females were removed from analysis due to missing values; three males and eight females were excluded because their RTs proved to be outliers. Thus, the final sample comprised 107 participants. All were native speakers of Hebrew, with normal or corrected‐to‐normal vision and no history of neurologically based impairments, such as attention‐deficit/hyperactivity disorder or learning disabilities (e.g., dyslexia and dyscalculia). Prior to data collection, participants signed a consent form approved by the University of Haifa ethics committee (429/17).

### Questionnaires

Questionnaires were translated by the author into Hebrew (forward translation) and from Hebrew back to English (back translation) to ensure the validity of translations.

#### Math anxiety

The short Mathematics Anxiety Rating Scale (sMARS)^75^ is a 25‐item version of the widely used 98‐item Mathematics Anxiety Rating Scale (MARS).^76^ Participants respond to questions about how anxious they would feel during different everyday (e.g., “Reading a cash register receipt after you buy something”) and formal situations (e.g., “Studying for a math test”) on a 5‐point Likert scale from *not at all* to *very much*. The total score is obtained by summing each item rating, with increasing scores reflecting an increased level of math anxiety. Cronbach's alpha for the sMARS in our sample was 0.96.

#### Habitual use of reappraisal and suppression

We used the well‐known Emotion Regulation Questionnaire (ERQ)^77^ to measure trait tendencies to use reappraisal and suppression. The ERQ has acceptable validity and reliability.^77^ It includes 10 items, six measuring reappraisal frequency (e.g., “I control my emotions by changing the way I think about the situation I'm in”) and four measuring expressive suppression frequency (e.g., “I control my emotions by not expressing them”). Items are rated on a 7‐point Likert‐type response scale from *strongly disagree* to *strongly agree*. Higher scores on each scale indicate greater use of the corresponding strategy. The total score of the frequency of the use of each strategy is assessed by the average score of the relevant subscale of the ERQ. The coefficient alpha for the ERQ in our sample was 0.83.

### Emotional flanker task

**Figure 1:**
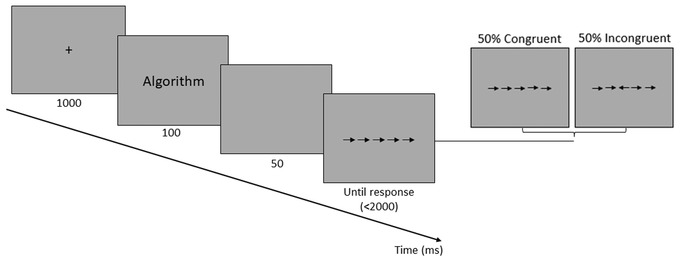
An example of a trial

The design of this task was based on a previous study.^17^ Figure 1 presents an example of a trial. At the beginning of each trial, a black colored square‐shaped fixation point was presented for 1000 milliseconds. Then, a Hebrew emotional word (i.e., math‐related word or word with negative valence) or pseudoword was presented for 100 milliseconds. The words were displayed in 32‐point Consolas bold font in black. A 150‐ms stimulus onset asynchrony preceded the flanker target; the target was displayed until the participant's response or at most for 2000 milliseconds. Flanker stimuli consisted of a line of five black arrows, with the middle arrow pointing in the same direction as (congruent stimuli) or a different direction from (incongruent stimuli) the flanking arrows. Each arrow subtended a visual angle of 1.5° from a viewing distance of 57 centimeters. The arrows were separated by 0.5°. In half of the trials, the flanker target was congruent, and in the other half, it was incongruent. Participants were asked to ignore the flanking arrows and to indicate the direction of the middle arrow by pressing a matching keyboard key (the D key with their left index finger to indicate a middle arrow pointing to the left, and the K key with their right index finger to indicate a middle arrow pointing to the right). A silver‐colored background was used throughout the experiment. The task was preceded by six practice trials in which accuracy feedback was given. Before starting the task, participants were instructed to look at the fixation cross and to respond as quickly and as accurately as possible to the flanker stimuli.

For the flanker task, we used 16 math‐related words (e.g., “algorithm”), 16 words with negative valence (e.g., “accident”), and 16 pseudowords with a standard morphological structure and no significant differences in length (*P = *0.48). Note that pseudowords were used as fillers in the experimental task and to produce the emotional distraction, an index of the level of executive control. The math‐related words and words with negative valence were taken from a database of emotional Hebrew words.^78^ While there was no significant difference between these words in frequency (*P = *0.44), math‐related words had higher math load (*M* = 2.63, *SD* = 0.21), *t*(15) = 50.54, *P* < 0.001) and elicited lower negative feelings (*M* = 1.88, *SD* = 0.15), *t*(22.17) = −25.33, *P* < 0.001) than words with negative valence (for mathematical load, *M* = 0.00, *SD* = 0.00; for negative feelings, *M* = 3.599, *SD* = 0.30). Each word was paired with a congruent flanker in one set, and with an incongruent flanker in the other set. The two sets were counterbalanced between participants, and the order of the trials was random. The task consisted of 96 trials (2 flanker congruities × 3 stimulus types × 16 trials per condition).

### Procedure

Due to COVID‐19 restrictions in Israel, participants completed the task and filled out the questionnaires online. Most participants (∼70%) were recruited through iPanel (iPanel.co.il; iPanel, Bnei‐Brak, Israel), an online Israeli pooling service, from June to September 2021. iPanel can deliver a representative sample of the adult Jewish population of Israel while adhering to the stringent standards of the European Society for Opinion and Marketing Research (ESOMAR). In addition, iPanel was evaluated by the Applied Statistical Laboratory of Hebrew University in Jerusalem and found to be highly accurate.^79^ Prospective participants who registered in iPanel and met the inclusion criteria received an invitation to participate; after giving their consent, they received vouchers by the survey company in exchange for their participation. An additional small proportion (∼30%) of participants was recruited through invitations posted on various student Internet groups and forums.^80^, ^81^ The posts described the study and invited those who met the inclusion criteria to participate by sending an email to the researchers’ email address. Prospective participants who registered this way received an invitation to participate and gave their consent; they received a monetary compensation equivalent to $10.

The study was administered via E‐Prime Go Software. The MARS questionnaire was presented first, followed by the emotional flanker task and the ERQ questionnaire. Note that this study was part of an ongoing larger study with additional cognitive tasks and emotional questionnaires.

### Statistical analyses

First, we divided the sample into two extreme math‐anxiety groups by plotting scores at ± 1 *SD* of the sample mean.^82^ This resulted in two groups: 17 slightly (*M* = 32.47, *SD* = 6.62) and 20 highly math‐anxious individuals (*M* = 94.95, *SD* = 8.24). Next, we conducted repeated measures analyses of covariance with a Bonferroni adjustment and post‐hoc comparisons using paired sample *t*‐tests with congruity (congruent, incongruent) and valence (math‐related words, words with negative valence) as within‐subject factors and math anxiety as a between‐subject variable to observe differences between highly and slightly math‐anxious individuals in the effects of emotional interference on the executive control of attention, while differentiating between trials preceded by words with negative valence and math‐related words. Effect sizes were tested using Cohen's *d*.^83^

Second, based on a previous study,^17^ to examine whether emotion regulation modulated the link between math anxiety and the (possible) deficient ability of executive control to reduce emotional distractions in incongruent situations, we assessed: (1) emotional distraction induced by irrelevant math‐related words by subtracting mean RTs in trials preceded by pseudowords from mean RTs in trials preceded by math‐related words; we called this variable emotional distraction induced by irrelevant math‐related words; and (2) emotional distraction induced by irrelevant words with negative valence by subtracting mean RTs in trials preceded by pseudowords from mean RTs in trials preceded by irrelevant words with negative valence.^17^ We named this variable emotional distraction induced by irrelevant words with negative valence. Emotional distractions in congruent trials induced by irrelevant math‐related and negative words were also computed in order to distinguish between general emotional interference (i.e., congruent trials) and the ability of executive control to reduce emotional distraction (i.e., incongruent trials).

Third, we performed correlative analysis followed by hierarchical linear regression analysis with emotional distractions induced by irrelevant math‐related and negative words in both congruent and incongruent trials as the dependent variables. These analyses were conducted to verify that math anxiety is related to specific math‐based emotional interference and emotion regulation did not modulate emotional distractions in general, only in high‐conflict conditions. Math anxiety was entered into the model as an independent variable followed by emotion regulation (reappraisal and suppression). The data and materials are available at http://osf.io/hjrck.

## Results

The final sample consisted of 107 adults (71 females; *M* = 28.27 years, *SD* = 4.32). Three males and eight females were excluded from analysis because their RTs were outliers (below 200 ms or above 1000 ms^17^). We calculated mean RTs in the various conditions (two congruency conditions × three word types). Descriptive statistics of the research variables are shown in Table 1.

**Table 1:** Descriptive statistics of research variables

	*M*	*SD*	Range
Math anxiety (sMARS)	63.31	21.04	25–108
ERQ reappraisal scale	4.66	1.26	1–7
ERQ suppression scale	3.44	1.53	1–7
*RT (ms) in congruent trials*			
Math‐related words	497.96	123.77	297.85–995.27
Words with negative valence	503.78	109.97	326.25–865.19
Pseudowords	515.33	124.67	318.42–889.75
*RT (ms) in incongruent trials*			
Math‐related words	549.22	133.01	318.13–954.38
Words with negative valence	537.05	136.03	284.13–903.86
Pseudowords	543.52	139.66	226.5–945.93

### Emotional effects on executive control of attention in math anxiety

**Figure 2:**
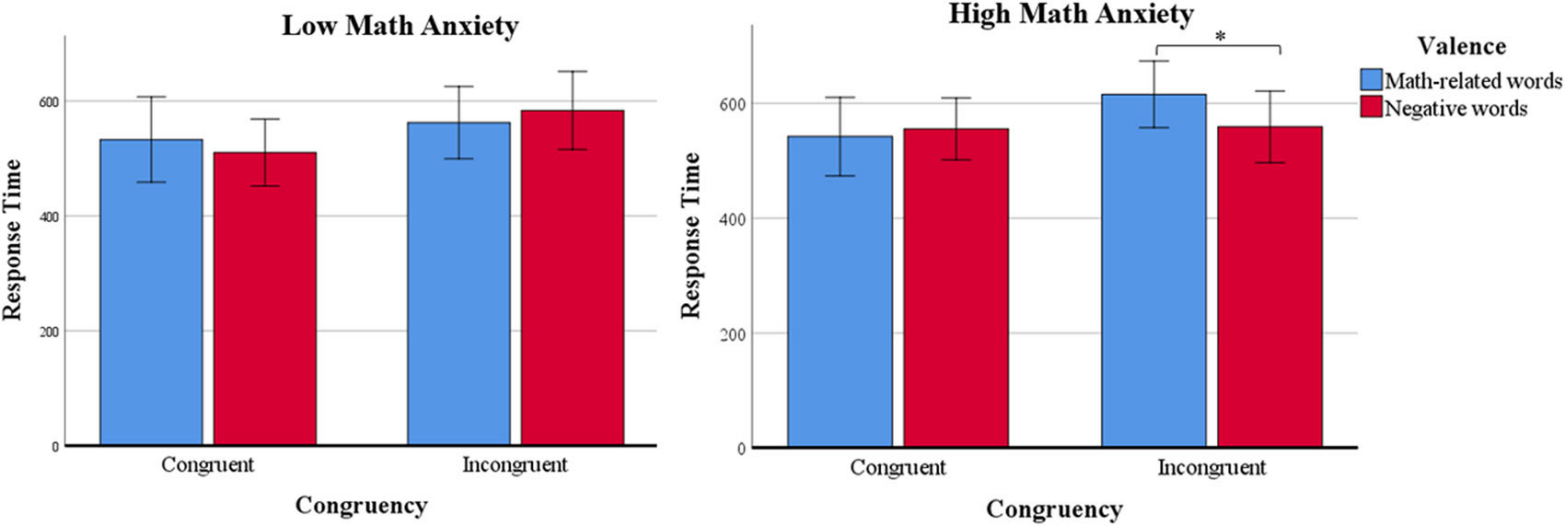
Differences between trials preceded by math‐related and negative words in congruent and incongruent trials among highly and slightly math‐anxious individuals

An investigation of differences in RTs between trials preceded by math‐related words and trials preceded by words with negative valence revealed a main effect of congruency, *F*(1,35) = 14.52, *P* = 0.001, *η* ^2^ *= *0.29, 95% CI: [494.15, 576.45]). Replicating previous results in the flanker task, participants were faster in congruent (*M* = 535.30, *SD* = 20.27) than in incongruent trials (*M* = 580.23, *SD* = 20.85). Importantly, there was a significant triple interaction between congruency (congruent, incongruent), valence (math‐related and negative words), and math anxiety (high, low) (*F*(1,35) = 4.69, *P* = 0.037, *η* ^2^ *= *0.12; see Fig. 2).

We then analyzed the simple double interactions between valence and congruency separately for highly and slightly math‐anxious individuals. Supporting our main hypothesis, in the slightly math‐anxious group, the interaction between valence and congruency was not significant (*P* = 0.30). In contrast, in the highly math‐anxious group, this double interaction was significant (*F*(1,19) = 4.37, *P* = 0.05, *η* ^2^ *= *0.19). Analysis of the highly math‐anxious group showed that in the incongruent trials only, there were significant differences in RTs for trials preceded by math‐related stimuli and trials preceded by words with negative valence (*t*(19) = 2.75, *P* = 0.01, Cohen's *d = *0.51, 95% CI: [13.56, 99.48]), indicating faster responses in incongruent trials preceded by words with negative valence (*M* = 559.07, *SD* = 142.62) than those preceded by math‐related words (*M* = 615.59, *SD* = 137.50). Meanwhile, in the congruent trials, this group showed no significant differences in RTs between trials preceded by math‐related stimuli and trials preceded by words with negative valence (*P = *0.52).

### Reappraisal as modulator of links between math anxiety and executive control of attention

**Figure 3:**
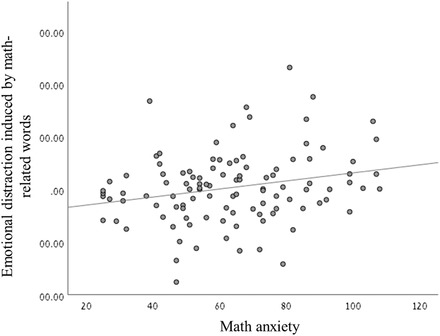
Scatterplot of correlation between math anxiety and emotional distraction induced by irrelevant math‐related words in incongruent trials

The correlation matrix is presented in Table 2. As expected, math anxiety was positively linked to emotional distraction induced by irrelevant math‐related words (mean RTs in trials preceded by math‐related words minus mean RTs in trials preceded by pseudowords) in the incongruent trials (*r* = 0.24, *P* = 0.01, 95% CI: [0.05, 0.43]; see Fig. 3), but not in congruent trials (*P* = 0.72). In addition, math anxiety was not associated with emotional distractions induced by irrelevant words with negative valence (i.e., mean RTs in trials preceded by negative words minus mean RTs in trials preceded by pseudowords) either in congruent (*P* = 0.31) or in incongruent trials (*P* = 0.31). These findings indicate that math anxiety is associated with reduced ability of executive control of attention to reduce emotional interference of math‐related information (i.e., in incongruent situations).

**Table 2:** Correlation matrix of research variables

Variable	1	2	3	4
1. sMARS				
*Emotional distractions in congruent trials*				
2. Induced by irrelevant math‐related words	0.04			
3. Induced by irrelevant words with negative valence	0.10	0.64**		
*Emotional distractions in incongruent trials*				
4. Induced by irrelevant math‐related words	0.24*	0.53**	0.47**	
5. Induced by irrelevant words with negative valence	0.10	0.52**	0.36**	0.78*

Note: Emotional distraction induced by irrelevant math‐related words = RTs in trials preceded by math‐related words minus RTs in trials preceded by pseudowords. Emotional distraction induced by irrelevant words with negative valence = RTs in trials preceded by words with negative valence minus RTs in trials preceded by pseudowords.

* *P* < 0.025.

** *P* < 0.001.

To examine our main question on relations between math anxiety, emotion regulation, and executive control of attention, we built multiple two‐stage hierarchical linear regression models with emotional distraction variables (i.e., emotional distractions induced by math‐related and negative words in congruent and incongruent trials) as dependent variables. Math anxiety was entered into the model at stage one and emotion regulation variables (reappraisal and suppression) were entered at stage two. Each analysis was examined for problems with multicollinearity using the variance inflation factor, but none of the analyses revealed significant problems (largest variance inflation factor = 1.02; see Refs. 84 and 85).

**Figure 4:**
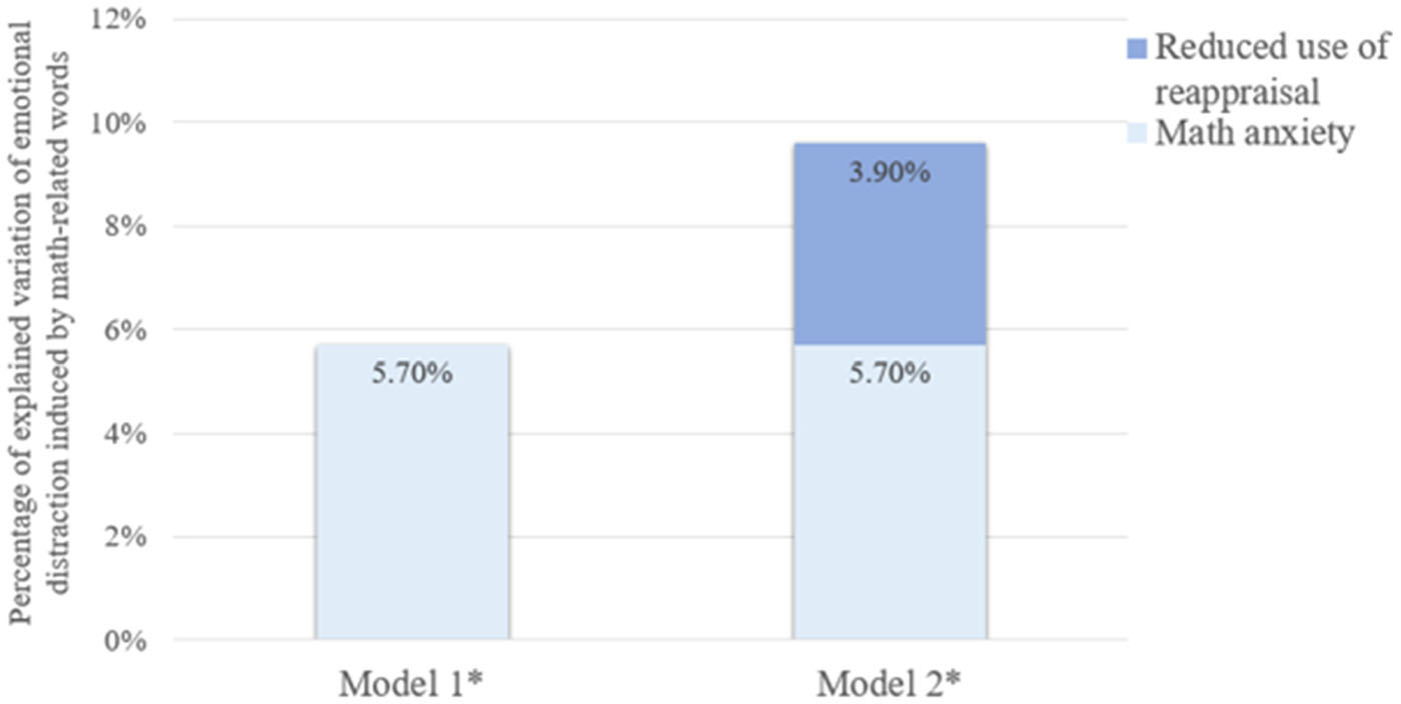
Increased math anxiety and reduced use of reappraisal predict emotional distraction induced by irrelevant math‐related words in incongruent trials

The regression statistics are reported in Table 3. The main analysis included emotional distractions in incongruent trials as the dependent variables. Results showed that math anxiety significantly explained 5.7% of the variance in emotional distraction induced by irrelevant math‐related words (*F*(1,105) = 6.31, *P* = 0.01, see Table 3A). In the next step, math anxiety (positively) and reappraisal (negatively) explained 9.6% of the variance (*F*(3,103) = 3.64, *P* = 0.02, see Table 3A). That is, consistent with our second hypothesis, increased math anxiety together with decreased tendencies to use reappraisal in everyday situations were associated with lower abilities to control emotional distraction induced by irrelevant math‐related words in the high‐conflict condition (i.e., incongruent trials; see Fig. 4).

**Table 3:** Summary of hierarchical regression analyses to predict emotional distractions

	Model 1					Model 2				
	*Β*	*95% CI*		*SE B*	*ẞ*	*Β*	*95% CI*		*SE B*	*ẞ*
Variables		*LL*	*UL*				*LL*	*UL*		
*3A. Emotional distraction induced by irrelevant math‐related words in incongruent trials*										
Math anxiety	1.53	0.32	2.73	0.61	0.24**	1.54	0.34	2.74	0.60	0.24**
Reappraisal						–21.21	–41.25	–1.16	10.11	–0.20*
Suppression						3.56	–12.96	20.08	8.33	0.04
*3B. Emotional distraction in incongruent trials induced by irrelevant words with negative valence*										
Math anxiety	0.61	–0.57	1.79	0.60	0.09	0.57	–0.60	1.74	0.59	0.09
Reappraisal						–21.74	–41.28	–2.21	9.85	–0.21
Suppression						–3.65	–19.76	12.45	8.12	–0.04
*3C. Emotional distraction in congruent trials induced by irrelevant math‐related words*										
Math anxiety	0.17	–0.77	1.12	0.48	0.04	0.20	–0.74	1.14	0.47	0.04
Reappraisal						–14.55	–30.27	1.17	7.93	–0.18
Suppression						5.36	–7.60	18.32	6.53	0.08
*3D. Emotional distraction in congruent trials induced by irrelevant words with negative valence*										
sMARS	0.50	–0.47	1.4	0.49	0.10	0.48	–0.50	1.46	0.49	0.10
Reappraisal						–7.66	–24.05	8.74	8.27	–0.09
Suppression						–1.25	–14.76	12.27	6.81	–0.02

Abbreviations: LL, lower limit of confidence interval; UL, upper limit of confidence interval.

* *P* < 0.05, ^**^ *P* < 0.025.

The contribution of suppression to the regression model was not significant (*P* = 0.67). Math anxiety and emotion regulation did not predict emotional distraction induced by irrelevant words with negative valence (*P* = 0.10; see Table 3B).

We conducted additional analyses in which emotional distractions in congruent trials served as the dependent variables. These analyses indicated that math anxiety and emotion regulation did not predict emotional distraction induced by irrelevant math‐related words (*P* = 0.27; see Table 3C) or words with negative valence (*P* = 0.59; see Table 3D), verifying that emotion regulation did not modulate emotional distractions in general, only when executive control of attention was required.

To summarize, our findings strongly suggest that when there is a need to deal with math‐related information, highly math‐anxious individuals’ executive control of attention to reduce emotional interference is disrupted. However, increased use of reappraisal in everyday situations can modulate the link between emotion and executive functions in math anxiety.

## Discussion

We innovatively examined the links between frequent use of emotion regulation strategies (i.e., reappraisal and suppression) and executive control in math anxiety. The findings demonstrated: (1) math‐anxious individuals find it difficult to control the emotional distraction caused by the exposure to numerical stimuli, even as simple as math‐related words, but this difficulty does not occur when words with negative valence are presented to them; and (2) the ability of the executive process to reduce math‐based emotional distractions is higher when math anxiety levels are lower but is even higher when math‐anxious individuals use reappraisal strategies in everyday situations.

Consistent with the literature,^3^, ^6^, ^7^ our findings suggest that math‐related information is perceived as threatening among highly but not slightly math‐anxious individuals. Previous work found that math‐related information was linked with negative affective valence, especially among females, regardless of math anxiety levels.^78^ Our study expands the literature on impairments in executive control of attention in math anxiety,^11^, ^12^, ^13^, ^23^, ^36^, ^37^ by showing that math anxiety is related to difficulties in reducing emotional distractions induced by math‐related information. An important finding was that reduced use of reappraisal predicted emotional distraction induced by math information in math anxiety. It seems that difficulties in emotion regulation, specifically reduced use of the adaptive reappraisal strategy in everyday situations, are not linked with general emotional distractions in math anxiety—only with emotional interferences induced by math information.

The study proposes a mechanism through which reappraisal‐focused interventions may lead to reduced math anxiety reactions^63^, ^64^ and improved math performance.^24^, ^67^ In our study, the ability of math‐anxious individuals to use reappraisal in daily life was associated with their ability to avoid heightened emotional reactions when encountering math‐related (i.e., threatening) information. Our finding that suppression was not related to task performance strengthens the idea of a specific link between the tendency to use reappraisal, rather than emotion regulation or executive functions.^21^, ^22^, ^46^, ^47^, ^49^ Reappraisal has been associated elsewhere with a decrease in the subjective experience of negative emotions^55^, ^56^, ^57^ and more adaptive responses to emotionally evocative events.^55^, ^56^, ^60^

The attentional control theory^34^ argues that difficulties in executive control mechanisms in math anxiety are due to external^9^, ^10^, ^38^ and internal^27^ emotional interference induced by math‐related information. In line with this theory, our findings demonstrate the emotional effects of math anxiety in a high‐conflict environment (i.e., incongruent situations) that required executive control. Specifically, these effects appeared when math information was presented to participants, not when stimuli with a negative valence without math‐related content were presented.

Given the interrelationships between emotion regulation and executive control of attention,^21^, ^22^ future research should examine the effects of training in executive mechanisms on the tendency of math‐anxious individuals to use reappraisal in everyday situations. The literature suggests the ability to use reappraisal successfully can be improved by cognitive control interventions.^46^, ^47^ In the field of math anxiety, more studies are required to: (1) shed light on the link between emotion regulation and executive control of attention to reduce math anxiety‐related emotional effects; and (2) develop more efficient interventions for math‐anxious individuals.

### Limitations

The study contributes to the understanding of the links between emotion regulation and the ability of executive control of attention to reduce emotional distractions in math anxiety but has some limitations. Recruiting participants via Internet and social networks may threaten the findings’ reliability and validity.^86^ However, Internet‐based data have shown high reliability, valid replicability, and theoretical consistency compared to data gathered in a traditional lab setting.^87^ In this study, the coefficient alphas for the math anxiety and emotion regulation tendencies questionnaires were 0.96 and 0.83, respectively. Moreover, most participants were recruited through an online Israeli pooling service found to be highly accurate by the Applied Statistical Laboratory of Hebrew University in Jerusalem.^79^

In addition, there is significant evidence that math performance is related to both math anxiety^25^, ^26^, ^27^ and executive control in a numerical context.^88^, ^89^ Therefore, the current study cannot account for possible influences of math performance on the relationships between math anxiety and executive control and further research is needed.

## Conclusion

The study illustrates, first, that math information is linked with negative affective valence among highly math‐anxious individuals, leading to greater difficulties in executive control of attention. These difficulties were not observed in our participants after the presentation of negative stimuli, only after the presentation of stimuli with math content. Second, difficulties in emotion regulation and reduced use of the adaptive reappraisal strategy in everyday situations predicted emotional distraction induced by math information in math anxiety. The study suggests an innovative mechanism through which reappraisal‐focused intervention reduces emotional reactions and improves performance among math‐anxious individuals, thus indicating a new approach to interventions for math anxiety.

## Author contributions

L.D.C. was the lead author in conceptualizing and designing the research, acquisition, analysis, and interpretation of data, and integration of the data analyzed. O.R. contributed to conception and design of the research, acquisition, analysis, and interpretation of data, and integration of the data analyzed. All authors participated in drafting the manuscript and revising its intellectual content; and approved the final version of the submitted manuscript.

## Competing interests

The authors declare no competing interests.

### Peer review

The peer review history for this article is available at: https://publons.com/publon/10.1111/nyas.14772

## References

[1] Hart, S.A. & C.M. Ganley . 2019. The nature of math anxiety in adults: prevalence and correlates. J. Numer. Cogn. 5: 122–139. 33842689 10.5964/jnc.v5i2.195 PMC8034611

[2] Dowker, A. , K. Bennett & L. Smith . 2012. Attitudes to mathematics in primary school children. Child Dev. Res. 2012: 124939.

[3] Kucian, K. , U. McCaskey , R.O. Tuura & M. von Aster . 2018. Neurostructural correlate of math anxiety in the brain of children. Transl. Psychiatry 8: 1–11. 30531959 10.1038/s41398-018-0320-6 PMC6288142

[4] Beilock, S.L. & E.A. Maloney . 2015. Math anxiety a factor in math achievement not to be ignored. Policy Insights Behav. Brain Sci. 2: 4–12.

[5] Ramirez, G. , S.T. Shaw & E.A. Maloney . 2018. Math anxiety: past research, promising interventions, and a new interpretation framework. Educ. Psychol. 53: 245–264.

[6] Moustafa, A.A. , A. Porter & A.M. Megreya . 2020. Mathematics anxiety and cognition: an integrated neural network model. Rev. Neurosci. 31: 287–296‏. 31730536 10.1515/revneuro-2019-0068

[7] Pizzie, R.G. & D.J. Kraemer . 2017. Avoiding math on a rapid timescale: emotional responsivity and anxious attention in math anxiety. Brain Cogn. 118: 100–107. 28826050 10.1016/j.bandc.2017.08.004

[8] Ashkenazi, S. 2018. Intentional and automatic processing of numerical information in mathematical anxiety: testing the influence of emotional priming. Cogn. Emot. 32: 1700–1707. 29400601 10.1080/02699931.2018.1435504

[9] Rubinsten, O. , H. Eidlin , H. Wohl & O. Akibli . 2015. Attentional bias in math anxiety. Front. Psychol. 6: 1539. 26528208 10.3389/fpsyg.2015.01539 PMC4607867

[10] Suárez‐Pellicioni, M. , M.I. Núñez‐Peña & A. Colomé . 2015. Attentional bias in high math‐anxious individuals: evidence from an emotional Stroop task. Front. Psychol. 6: 1577. 26539137 10.3389/fpsyg.2015.01577 PMC4609828

[11] Mammarella, I.C. , S. Caviola , D. Giofrè & E. Borella . 2018. Separating math from anxiety: the role of inhibitory mechanisms. Appl. Neuropsychol. Child 7: 342–353. 28682117 10.1080/21622965.2017.1341836

[12] Justicia‐Galiano, M.J. , S. Pelegrina , M.T. Lechuga , *et al*. 2016. Math anxiety and its relationship to inhibitory abilities and perceived emotional intelligence. Anal. Psicol. 32: 125–131.

[13] Liu, J. , J. Li , W. Peng , *et al*. 2019. EEG correlates of math anxiety during arithmetic problem solving: implication for attention deficits. Neurosci. Lett. 703: 191–197. 30928479 10.1016/j.neulet.2019.03.047

[14] Banich, M.T. 2009. Executive function: the search for an integrated account. Curr. Dir. Psychol. Sci. 18: 89–94.

[15] Miller, E.K. & J.J. Cohen . 2001. An integrative theory of prefrontal cortex function. Annu. Rev. Neurosci. 24: 167–202. 11283309 10.1146/annurev.neuro.24.1.167

[16] Adam, R. , S. Schönfelder , J. Forneck & M. Wessa . 2014. Regulating the blink: cognitive reappraisal modulates attention. Front. Psychol. 5: 143. 24596568 10.3389/fpsyg.2014.00143 PMC3931308

[17] Cohen, N. , A. Henik & N. Moyal . 2012. Executive control attenuates emotional effects—for high reappraisers only? Emotion 12: 970–979. 22251044 10.1037/a0026890

[18] Gross, J.J. 2015. Emotion regulation: current status and future prospects. Psychol. Inq. 26: 1–26.

[19] McRae, K. & J.J. Gross . 2020. Introduction: emotion regulation. Emotion 20: 1–9. 31961170 10.1037/emo0000703

[20] Buhle, J.T. , J.A. Silvers , T.D. Wager , *et al*. 2014. Cognitive reappraisal of emotion: a meta‐analysis of human neuroimaging studies. Cereb. Cortex 24: 2981–2990. 23765157 10.1093/cercor/bht154 PMC4193464

[21] Quinn, M.E. & J. Joormann . 2020. Executive control under stress: relation to reappraisal ability and depressive symptoms. Behav. Res. Ther. 131: 103634. 32387887 10.1016/j.brat.2020.103634 PMC7336894

[22] Wante, L. , A. Mezulis , M.L. Van Beveren & C. Braet . 2017. The mediating effect of adaptive and maladaptive emotion regulation strategies on executive functioning impairment and depressive symptoms among adolescents. Child Neuropsychol. 23: 935–953. 27535347 10.1080/09297049.2016.1212986

[23] Van den Bussche, E. , K. Vanmeert , B. Aben & D. Sasanguie . 2020. Too anxious to control: the relation between math anxiety and inhibitory control processes. Sci. Rep. 10: 19922. 33199798 10.1038/s41598-020-76920-7 PMC7670467

[24] Pizzie, R.G. , C.L. McDermott , T.G. Salem & D.J. Kraemer . 2020. Neural evidence for cognitive reappraisal as a strategy to alleviate the effects of math anxiety. Soc. Cogn. Affect. Neurosci. 15: 1271–1287. 33258958 10.1093/scan/nsaa161 PMC7759208

[25] Ashcraft, M.H. & A.M. Moore . 2009. Mathematics anxiety and the affective drop in performance. J. Psychoed. Assess. 27: 197–205.

[26] Dowker, A. , A. Sarkar & C.Y. Looi . 2016. Mathematics anxiety: what have we learned in 60 years? Front. Psychol. 7: 508. 27199789 10.3389/fpsyg.2016.00508 PMC4842756

[27] Chang, H. & S.L. Beilock . 2016. The math anxiety–math performance link and its relation to individual and environmental factors: a review of current behavioral and psychophysiological research. Curr. Opin. Behav. Sci. 10: 33–38.

[28] Skagerlund, K. , R. Östergren , D. Västfjäll & U. Träff . 2019. How does mathematics anxiety impair mathematical abilities? Investigating the link between math anxiety, working memory, and number processing. PLoS One 14: e0211283. 30682125 10.1371/journal.pone.0211283 PMC6347150

[29] ‏Passolunghi, M.C. , E. Cargnelutti & S. Pellizzoni . 2019. The relation between cognitive and emotional factors and arithmetic problem‐solving. Educ. Stud. Math. 100: 271–290.

[30] Ahmed, W. 2018. Developmental trajectories of math anxiety during adolescence: associations with STEM career choice. J. Adolesc. 67: 158–166. 29975882 10.1016/j.adolescence.2018.06.010

[31] Woloshin, S. , L.M. Schwartz , M. Moncur , *et al*. 2001. Assessing values for health: numeracy matters. Med. Decis. Making 21: 382–390. 11575488 10.1177/0272989X0102100505

[32] Skagerlund, K. , T. Lind , C. Strömbäck , *et al*. 2018. Financial literacy and the role of numeracy–how individuals’ attitude and affinity with numbers influence financial literacy. J. Behav. Exp. Econ. 74: 18–25.

[33] Ritchie, S.J. & T.C. Bates . 2013. Enduring links from childhood mathematics and reading achievement to adult socioeconomic status. Psychol. Sci. 24: 1301–1308. 23640065 10.1177/0956797612466268

[34] Eysenck, M. , N. Derakshan , R. Santos & M.G. Calvo . 2007. Anxiety and cognitive performance: attentional control theory. Emotion 7: 336–353. 17516812 10.1037/1528-3542.7.2.336

[35] Eysenck, M.W. & M.G. Calvo . 1992. Anxiety and performance: the processing efficiency theory. Cogn. Emot. 6: 409–434.

[36] Pletzer, B. , M. Kronbichler , H.C. Nuerk & H.H. Kerschbaum . 2015. Mathematics anxiety reduces default mode network deactivation in response to numerical tasks. Front. Hum. Neurosci. 9: 202. 25954179 10.3389/fnhum.2015.00202 PMC4404831

[37] ‏Suárez‐Pellicioni, M. , M.I. Núñez‐Peña & A. Colomé . 2014. Reactive recruitment of attentional control in math anxiety: an ERP study of numeric conflict monitoring and adaptation. PLoS One 9: e99579. 24918584 10.1371/journal.pone.0099579 PMC4053379

[38] Huang, B. , X. Zhao , H. Li , *et al*. 2019. Arithmetic skill may refine the performance of individuals with high math anxiety, especially in the calculation task: an ERP study. Sci. Rep. 9: 13283. 31527669 10.1038/s41598-019-49627-7 PMC6746767

[39] Friedman, N.P. & T.W. Robbins . 2021. The role of prefrontal cortex in cognitive control and executive function. Neuropsychopharmacology 47: 72–89. 34408280 10.1038/s41386-021-01132-0 PMC8617292

[40] Korem, N. , L.D. Cohen & O. Rubinsten . 2022. The link between math anxiety and performance does not depend on working memory: a network analysis study. Conscious. Cogn. 100: 103298. 35217396 10.1016/j.concog.2022.103298

[41] Eriksen, B.A. & C.W. Eriksen . 1974. Effects of noise letters upon the identification of a target letter in a nonsearch task. Atten. Percept. Psychophys. 16: 143–149.‏

[42] Norman, D.A. & T. Shallice . 1980. Attention to action: willed and automatic control of behavior. Technical Report No. 8006.‏‏ University of California.

[43] West, R. & C. Alain . 2000. Effects of task context and fluctuations of attention on neural activity supporting performance of the Stroop task. Brain Res. 873: 102–111. 10915815 10.1016/s0006-8993(00)02530-0

[44] Hofman, D. & D.J. Schutter . 2012. Asymmetrical frontal resting‐state beta oscillations predict trait aggressive tendencies and behavioral inhibition. Soc. Cogn. Affect. Neurosci. 7: 850–857. 22016441 10.1093/scan/nsr060 PMC3475360

[45] Jensen, O. , J. Kaiser & J.P. Lachaux . 2007. Human gamma‐frequency oscillations associated with attention and memory. Trends Neurosci. 30: 317–324. 17499860 10.1016/j.tins.2007.05.001

[46] Cohen, N. & N. Mor . 2018. Enhancing reappraisal by linking cognitive control and emotion. Clin. Psychol. Sci. 6: 155–163.

[47] Molavi, P. , S. Aziziaram , S. Basharpoor , *et al*. 2020. Repeated transcranial direct current stimulation of dorsolateral‐prefrontal cortex improves executive functions, cognitive reappraisal emotion regulation, and control over emotional processing in borderline personality disorder: a randomized, sham‐controlled, parallel‐group study. J. Affect. Disord. 274: 93–102. 32469838 10.1016/j.jad.2020.05.007

[48] Scherer, K.R. 1994. Emotion serves to decouple stimulus and response. In The Nature of Emotion: Fundamental Questions P. Ekman & R.J. Davidson, Eds.:. 127–130. New York: Oxford University Press.

[49] Scult, M.A. , A.R. Knodt , J.R. Swartz , *et al*. 2017. Thinking and feeling: individual differences in habitual emotion regulation and stress‐related mood are associated with prefrontal executive control. Clin. Psychol. Sci. 5: 150–157. 28191365 10.1177/2167702616654688 PMC5298870

[50] Ochsner, K.N. & J.J. Gross . 2005. The cognitive control of emotion. Trends Cogn. Sci. 9: 242–249. 15866151 10.1016/j.tics.2005.03.010

[51] Dreisbach, G. & K. Fröber . 2019. On how to be flexible (or not): modulation of the stability–flexibility balance. Curr. Dir. Psychol. Sci. 28: 3–9.

[52] Sutton, T.M. & C. Lutz . 2019. Attentional capture for emotional words and images: the importance of valence and arousal. Can. J. Exp. Psychol. 73: 47–54. 30124315 10.1037/cep0000154

[53] Cohen, N. & K.N. Ochsner . 2018. From surviving to thriving in the face of threats: the emerging science of emotion regulation training. Curr. Opin. Behav. Sci. 24: 143–155. 31187051 10.1016/j.cobeha.2018.08.007 PMC6559737

[54] Bigman, Y.E. , G. Sheppes & M. Tamir . 2017. When less is more: effects of the availability of strategic options on regulating negative emotions. Emotion 17: 993–1006. 28277712 10.1037/emo0000303

[55] Balzarotti, S. , V. Chiarella & M.R. Ciceri . 2017. Individual differences in cognitive reappraisal predict emotional experience prior to achievement situations. J. Individ. Diff. 38: 144–154.

[56] Goldin, P.R. , C.A. Moodie & J.J. Gross . 2019. Acceptance versus reappraisal: behavioral, autonomic, and neural effects. Cogn. Affect. Behav. Neurosci. 19: 927–944. 30656602 10.3758/s13415-019-00690-7

[57] Troy, A.S. , A.J. Shallcross , A. Brunner , *et al*. 2018. Cognitive reappraisal and acceptance: effects on emotion, physiology, and perceived cognitive costs. Emotion 18: 58–74. 29154585 10.1037/emo0000371 PMC6188704

[58] McRae, K. , B. Ciesielski & J.J. Gross . 2012. Unpacking cognitive reappraisal: goals, tactics, and outcomes. Emotion 12: 250–255. 22148990 10.1037/a0026351

[59] Schönfelder, S. , P. Kanske , J. Heissler & M. Wessa . 2014. Time course of emotion‐related responding during distraction and reappraisal. Soc. Cogn. Affect. Neurosci. 9: 1310–1319. 23988760 10.1093/scan/nst116 PMC4158366

[60] Sammy, N. , P.A. Anstiss , L.J. Moore , *et al*. 2017. The effects of arousal reappraisal on stress responses, performance and attention. Anxiety Stress Coping 30: 619–629. 28535726 10.1080/10615806.2017.1330952

[61] Liu, J.J. , N. Ein , J. Gervasio & K. Vickers . 2019. The efficacy of stress reappraisal interventions on stress responsivity: a meta‐analysis and systematic review of existing evidence. PLoS One 14: e0212854. 30811484 10.1371/journal.pone.0212854 PMC6392321

[62] Jamieson, J.P. , W.B. Mendes , E. Blackstock & T. Schmader . 2010. Turning the knots in your stomach into bows: reappraising arousal improves performance on the GRE. J. Exp. Soc. Psychol. 46: 208–212. 20161454 10.1016/j.jesp.2009.08.015 PMC2790291

[63] Jamieson, J.P. , B.J. Peters , E.J. Greenwood & A.J. Altose . 2016. Reappraising stress arousal improves performance and reduces evaluation anxiety in classroom exam situations. Soc. Psychol. Personal. Sci. 7: 579–587.

[64] Pizzie, R. & D.J. Kraemer . 2021. The association between emotion regulation, physiological arousal, and performance in math anxiety. Front. Psychol. 12: 639448. 34045991 10.3389/fpsyg.2021.639448 PMC8144633

[65] Brooks, A.W. 2014. Get excited: reappraising pre‐performance anxiety as excitement. J. Exp. Psychol. 143: 1144–1158. 10.1037/a0035325 24364682

[66] Ramirez, G. & S.L. Beilock . 2011. Writing about testing worries boosts exam performance in the classroom. Science 331: 211–213. 21233387 10.1126/science.1199427

[67] Rozek, C.S. , G. Ramirez , R.D. Fine & S.L. Beilock . 2019. Reducing socioeconomic disparities in the STEM pipeline through student emotion regulation. Proc. Natl. Acad. Sci. USA 116: 1553–1558. 30642965 10.1073/pnas.1808589116 PMC6358706

[68] Sarkar, A. , A. Dowker & R.C. Kadosh . 2014. Cognitive enhancement or cognitive cost: trait‐specific outcomes of brain stimulation in the case of mathematics anxiety. J. Neurosci. 34: 16605–16610. 25505313 10.1523/JNEUROSCI.3129-14.2014 PMC4261089

[69] Passolunghi, M.C. , C. De Vita & S. Pellizzoni . 2020. Math anxiety and math achievement: the effects of emotional and math strategy training. Dev. Sci. 23: e12964. 32159906 10.1111/desc.12964

[70] Goldin, P.R. , K. McRae , W. Ramel & J.J. Gross . 2008. The neural bases of emotion regulation: reappraisal and suppression of negative emotion. Biol. Psychiatry 63: 577–586. 17888411 10.1016/j.biopsych.2007.05.031 PMC2483789

[71] Niendam, T.A. , A.R. Laird , K.L. Ray , *et al*. 2012. Meta‐analytic evidence for a superordinate cognitive control network subserving diverse executive functions. Cogn. Affect. Behav. Neurosci. 12: 241–268. 22282036 10.3758/s13415-011-0083-5 PMC3660731

[72] McRae, K. , S.E. Jacobs , R.D. Ray , *et al*. 2012. Individual differences in reappraisal ability: links to reappraisal frequency, well‐being, and cognitive control. J. Res. Pers. 46: 2–7.

[73] Okon‐Singer, H. , D.M. Stout , M.D. Stockbridge , *et al*. 2017. The interplay of emotion and cognition. In The Nature of Emotion: Fundamental Questions. A.S. Fox , R.C. Lapate , A.J. Shackman & R.J. Davidson , Eds.: 181–186. New York: Oxford University Press.

[74] Musch, J. & U.D. Reips . 2000. A brief history of Web experimenting. In Psychological Experiments on the Internet. M.H. Birnbaum , Ed.: 61–87. Academic Press.

[75] Alexander, L. & C.R. Martray . 1989. The development of an abbreviated version of the Mathematics Anxiety Rating Scale. Meas. Eval. Couns. Dev. 22: 143–150.

[76] Richardson, F.C. & R.M. Suinn . 1972. The Mathematics Anxiety Rating Scale: psychometric data. J. Counsel. Psychol. 19: 551–554.

[77] Gross, J.J. & O.P. John . 2003. Individual differences in two emotion regulation processes: implications for affect, relationships, and well‐being. J. Pers. Soc. Psychol. 85: 348–362. 12916575 10.1037/0022-3514.85.2.348

[78] Daches Cohen, L. , L.L. Yavin & O. Rubinsten . 2021. Females' negative affective valence to math‐related words. Acta Psychol. 217: 103313. 10.1016/j.actpsy.2021.103313 33930625

[79] Nirel, N. 2011. Letter from the applied statistic laboratory. Department of Statistics, Hebrew University (Jerusalem) to the iPanel company.

[80] Roy, D. , S. Tripathy , S.K. Kar , *et al*. 2020. Study of knowledge, attitude, anxiety & perceived mental healthcare need in Indian population during COVID‐19 pandemic. Asian J. Psychiatr. 51: 1–7. 10.1016/j.ajp.2020.102083 PMC7139237 32283510

[81] Zsido, A. , N. Arato , O. Inhof , *et al*. 2018. Short versions of two specific phobia measures: the snake and the spider questionnaires. J. Anxiety Disord. 54: 11–16. 29306023 10.1016/j.janxdis.2017.12.002

[82] Ramirez, G. , H. Chang , E.A. Maloney , *et al*. 2016. On the relationship between math anxiety and math achievement in early elementary school: the role of problem solving strategies. J. Exp. Child Psychol. 141: 83–100. 26342473 10.1016/j.jecp.2015.07.014

[83] Cohen, J. 1988. Statistical Power Analysis for the Behavioral Sciences. Hillsdale, NJ: Erlbaum.

[84] Coakes, S.J. 2005. SPSS: Analysis without Anguish Using: Version 12.0 for Windows. Milton, QLD: John Wiley & Sons.

[85] Hair, J.F. , R.E. Anderson , R.L. Tatham & W.C. Black . 1998. Multivariate Data Analysis (5th ed.). Upper Saddle River, NJ: Prentice Hall International.

[86] Gosling, S.D. , S. Vazire , S. Srivastava & O.P. John . 2004. Should we trust web‐based studies? A comparative analysis of six preconceptions about internet questionnaires. Am. Psychol. 59: 93–104. 14992636 10.1037/0003-066X.59.2.93

[87] Germine, L. , K. Nakayama , B.C. Duchaine , *et al*. 2012. Is the Web as good as the lab? Comparable performance from Web and lab in cognitive/perceptual experiments. Psychon. Bull. Rev. 19: 847–857. 22829343 10.3758/s13423-012-0296-9

[88] Cragg, L. , S. Keeble , S. Richardson , *et al*. 2017. Direct and indirect influences of executive functions on mathematics achievements. Cognition 162: 12–26. 28189034 10.1016/j.cognition.2017.01.014

[89] Wilkey, E.D. , C. Pollack & G.R. Price . 2020. Dyscalculia and typical math achievement are associated with individual differences in number‐specific executive function. Child Dev. 91: 596–619. 30597527 10.1111/cdev.13194 PMC8183686

